# Upregulation of CENPM promotes hepatocarcinogenesis through mutiple mechanisms

**DOI:** 10.1186/s13046-019-1444-0

**Published:** 2019-11-08

**Authors:** Yusha Xiao, Rahmathullah Mohamed Najeeb, Dong Ma, Kang Yang, Qiu Zhong, Quanyan Liu

**Affiliations:** 1 Department of General Surgery, Zhongnan Hospital of Wuhan University, Wuhan, 430071 Hubei People’s Republic of China; 2 Department of Urology, Renmin Hospital of Wuhan University, Hubei, China

**Keywords:** Hepatocellular carcinoma (HCC), The Cancer genome atlas (TCGA), Centromere protein M (CENPM), Bioinformatics, P53, miR-1270

## Abstract

**Background:**

Hepatocellular carcinoma (HCC) still remains a dominating medical challenge in early diagnosis and clinical therapy. Centromere protein M (CENPM) has been proved to be over-expressed in HCC tissues, but carcinogenic mechanism of CENPM contributing to liver cancer is poorly understood.

**Methods:**

In this study, we first explored mRNA and protein levels of CENPM in HCC samples, matching adjacent non-tumor tissues and six hepatoma cell lines by polymerase chain reaction (PCR), western blotting and immunohistochemistry (IHC). Clinical data of HCC patients downloaded from The Cancer Genome Atlas (TCGA) were also analyzed. The character of CENPM concerned with HCC progression through several functional experimentations in vitro and in vivo was researched. Bioinformatics was carried out to further discover biological functions of CENPM.

**Results:**

CENPM was positively up-regulated in HCC and connected with a poor prognosis. Silencing CENPM repressed cell proliferation in vivo and in vitro, and knock-down CENPM inhibited cell migration and invasion. Additionally, depletion of CENPM can promote cell apoptosis and arrested cell cycle. Furthermore, single-gene gene set enrichment analysis (GSEA) analysis indicated that CENPM was linked to the P53 signaling pathway and cell cycle pathway, and our research supported this prediction. Finally, we also found that miR-1270 was a negative regulator and participated in post-transcriptional regulation of CENPM, and hepatitis B virus X protein (HBx) can promote hepatocellular carcinoma by suppressing miR1270.

**Conclusion:**

CENPM was closely associated with HCC progression and it could be considered as a new possible biomarker along with a therapeutic target for HCC.

## Background

Hepatocellular carcinoma (HCC) is the sixth most widespread cancer throughout the world, ranking second in tumor-related mortality [1, 2]. The number of newly diagnosed HCC worldwide is 780,000 every year, which is almost equal to the annual incidence of 740,000 death [3, 4]. And 80% cases of diagnosed HCC suffered from hepatitis B and C virus [5–7]. Due to the difficulties in the early diagnosis and lack of effective nonsurgical treatment methods, patients of HCC have a poor prognosis and most of them are not eligible for radical resection when they first diagnosed HCC [2, 8]. Even so, surgical resection still holds dominant position [9]. However, since most patients with HCC are already at the middle or advanced stage when diagnosed, the postoperative survival rate of liver cancer is not high [2]. In the United States, the five-year survival rate of patients with HCC is only 18% [10]. Therefore, novel biomakers as well as better understanding the molecular mechanisms and biological process of liver cancer are crucial for both the research and development of effective anticancer drugs and early diagnosis of HCC.

Previous study showed that aberrant function of proteins involved in chromosome separation could induce aneuploidy which was found in many types of cancer [11]. Centromere protein M (CENPM), also referred as proliferation associated nuclear element 1 (PANE1), is a kind of kinetochores protein which was detected in mouse mammary epithelium [12] and participated in affecting chromosome separation in the progress of cellular division [13]. CENPM was not only kinetochores protein that associated with microtubules to regulate chromosome segregation during cell division, but also took part in the biological function of the cell cycle [14, 15]. The expression of CENPM and centromere assembly should be controlled closely during the cell cycle, and mistakes in this regulation may result in aneuploidy. Besides, the other CENPM transcript encoded a leukocyte antigen that was mainly expressed in B-lymphoid cells to participate in the immune response [16]. Numerous researches pointed out some genes, including CENPA [17], CENPE [18], and CENPF [19, 20], which were homogeneous and closely correlated with tumors, and the results manifested that high-expression of centromere protein family could have a significant impact on the proliferation and invasion of tumors.

In this study, we discovered that CENPM was overexpressed in HCC samples and CENPM upregulation was closely concerned with a poor prognosis. Additionally, we detected that CENPM inhibited cell apoptosis and promoted cell cycle progression via affecting the P53 signal pathway. In addition, this research also demonstrated that miR1270 may be involved in posttranscriptional regulation of CENPM and HBx can decrease miR-1270 expression to upregulate CENPM to further promote HCC.

## Materials and methods

### Patients specimens and clinical data collection

Primary liver cancer tissues and corresponding adjacent not-tumor samples in 68 patients with HCC from Zhongnan Hospital of Wuhan University were collected. And specimens were immediately stockpiled at − 80 °C with an RNAlater solution (Invitrogen, USA). Patients were all confirmed HCC by histopathological examination after hepatectomy. Informed consent attained from patients who participated in this research. Besides, the clinical data of patients were acquired from the electronic medical record of general surgery department of Zhongnan Hospital. And this study also got approval from the ethics committee of Zhongnan Hospital.

### Cell culture

Human HCC cell lines including Huh7, SK-hep1, Hep3B, SMMC7721, HCCLM3, HepG2 and immortalized human liver cell line of HL-7702(L02) were purchased from the Cell Bank of Type Culture Collection (CBTCC, China). All cell lines were routinely supplied with high-glucose DMEM (Gibco, USA) containing 10% fetal bovine serum (Gibco, USA). All cells were nurtured in a rsbiotech at 37 °C with 5% CO2.

### Total RNA extraction and quantitative real-time PCR

Trizol reagent (Invitrogen, USA) was used for isolating total RNA from tumor tissues and cells. Reverse transcription of CENPM was accomplished using PrimeScript RT Master Mix (Takara, Japan). As for miRNA1270, the reverse transcription PCR was conducted by miRNA cDNA Synthesis Kit (ABM Inc., Canada). Quantitative real-time PCR was finished via SYBR qPCR Mix (Toyobo, Japan) and a CFX Connect Real- Time PCR Detection System (Bio-Rad, USA). The expression level of CENPM and miRNA1270 in HCC tissues and cell lines were evaluated using 2^-ΔΔCT^. The GAPDH and U6 level were regarded as an internal control. The primer sequences involved in this study were demonstrated in Additional file 1: Table S1.

### RNA interference, plasmid construction, lentiviral construction, and cell transfections

Human CENPM targeted small interfering RNAs (siRNAs) and miR-1270 mimics as well as inhibitors were designed and bought from GeneCreate (Wuhan, China). CENPM was inserted into pcDNA3.1 vector provided by GeneCreate (Wuhan, China), Lipofectamine 3000 reagent (Thermo Fisher Scientific, USA) and Lipofectamine 2000 reagent (Thermo Fisher Scientific, USA) were used for plasmid and small RNA transfection, respectively. Lentiviral small hairpin RNA (shRNA) expression vector targeting human CENPM gene (shRNA-CENPM) was constructed and synthesized by GeneCreate (Wuhan, China) and stably transfected into HCCLM3 cell line. A scrambled shRNA (shRNA-NC) was considered as the negative control for shRNA-CENPM. Lentivirus carrying full length HBx (HBx-FL) and lentiviral particles for negative control were constructed and purchased from GeneCopoeia (Wuhan, China) and stably transfected into Huh7 and HepG2 cell lines. The siRNA sequences applied to this research and primers designed for PCR of full-lenth HBx were presented in Additional file 1: Table S1.

### Immunohistochemistry (IHC) and immunofluorescence (IF)

For IHC, formalin-fixed tumor tissue sections were deparaffinized and rehydrated. Then heated at 105 °C for 10 min in a citric acid buffer (0.01 M) to antigen retrieval. The sliced tissues were later dealt with 3% hydrogen peroxide solution to block the endogenous peroxidase activity. Next, the slides were blocked with bovine serum albumin (BSA), then successively incubated with the primary antibody overnight at 4 °C and horseradish peroxidase (HRP)-conjugated secondary antibody. Immune complexes were detected through the standard substrate detection of HRP. Last, the slides were stained with hematoxylin and dehydration in graded alcohols and xylene.

For IF, 4% paraformaldehyde-fixed cells were permeabilized by 0.5% Triton X-100 and successively incubated with primary antibody and secondary antibody according to the product’s protocol. The slides were counterstained with DAPI and imaged with Olympus FV1000 (Tokyo, Japan).

### Cell proliferation assay

Huh7 and HepG2 cells were transfected with siRNA or control, then cell proliferation was conducted by CCK8 assays on the basis of supplier’s instructions. The transfected cells as described above were use for cell colony formation assay.Then colonies were fixed with 4% paraformaldehyde, stained by crystal violet solution and calculated.

### Cell migration and invasion assay

Cell migratory ability was assessed by scratch assay. After transfection with targeted-siRNA, 10^6^ cells were scattered into 6 well plates under serum-free conditions. The wounds were made via a 100ul plastic pipette tip. And the migration capability was measured after 24 or 48 h by the migrating distance. The cell invasion assay was conducted by a 24-well plate and a matrigel chamber (BD Biosciences, USA). Transfected cells were seeded in the upper chamber evenly without serum, and the culture medium supplemented with 15% FBS was added into the lower chamber of the 24-well plate as a inducer to trigger cell invasion. The formalin-fixed migrated cells on the subface of the matrigel chamber were stained by crystal violet and counted.

### Flow cytometry analysis

Huh7 and HepG2 cells were transfected with desired siRNA or control, then cell apoptosis assay were performed with the Annexin V-FITC/PI Apoptosis Detection Kit from the Beyotime Biotechnology (Shanghai, China). The cells were analyzed by flow cytometry (FACSCalibur flow cytometer; BD Biosciences, Franklin Lakes, NJ, USA).

The cells were stained with PI to conduct cell cycle analysis using cell cycle staining kit (Beyotime Biotechnology, China) according to the manufacturer’s protocol.

### Western blotting

Protein samples were extracted using RIPA cell lysis buffer with protease inhibitors and phosphatase inhibitors (Roche, Germany). The total protein quantity was measured by a BCA assay (Beyotime Biotechnology, China). Then protein samples were electrophoresed on 10% sodium dodecyl sulfate–polyacrylamide (SDS-PAGE) gels and subsequently transferred to polyvinylidene difluoride (PVDF) membranes, which were blocked with 5% skimmed milk and incubated at 4 °C overnight with targeted.

Primary antibodies on a rotating wheel. The membranes were washed three times and immediately following the incubation with corresponding HRP-conjugated secondary antibodies for 1 hour, proteins were finally developed utilizing Clarity Western ECL Substrate (Bio-Rad, USA). Explanations of antibodies involved in this research were demonstrated in Additional file 1: Table S2.

### Tumorigenesis assay in vivo

For xenograft experiments, five-week-old male BALB/c nude mice were bought from the Animal Center of the Chinese Academy of Medical Sciences (Beijing, China). HCCLM3 cells stably transfected with shRNA-CENPM or a shRNA-NC were subcutaneously injected into the armpits of 5 nude mice per group. CENPM silencing efficiency in HCCLM3 was verified to be significant (Additional file 3: Figure S2A). Tumor volumes of mice were measured every 5 days using a vernier caliper. Six weeks following the injection, the mice were killed to dissect and image the tumors.

To detect the intrahepatic tumorigenicity, an orthotopic HCC mouse model was established. First of all, the subcutaneous tumors of mice were implanted according to the methods mentioned above. Then tumors were collected when they reached 1 cm in diameter and cut into pieces with 1 mm^3^ in size under aseptic condition. After washing with phosphate buffer saline (PBS), the tumors were soaked in the serum-free medium. Subsequently, the nude mice were anesthetized, then made a midline abdominal incision to expose the liver, following one piece of tumors was implanted into the left liver lobe of every mouse. Finally, compression to stop bleeding and suturing abdominal incision. After 6 weeks, mice were examined by PET/CT conducted by Union Hospital PET Center of Tongji Medical College (Wuhan, China), then the mice were sacrificed to dissect liver tumor for subsequent experiments.

### Hematoxylin–eosin (HE) staining

The collected tumor specimens from liver of mice were fixed in 4% paraformaldehyde. Tissues were dehydrated, embedded in paraffin, and cut into slices. The sections were stained with hematoxylin and eosin and observed by light microscopy (Olympus, Japan).

### Dual luciferase reporter activity assay

The 3′UTR of CENPM, containing the binding site of miR-1270 and corresponding mutant sequences, was constructed into the pmirGLO vector and was cotransfected with miR-1270 mimics into Huh7 cells. Then dual luciferase reporter assay was performed by GeneCreate (Wuhan, China) with instructions of the Dual-Luciferase Reporter Assay system (Promega, Madison, USA). And firefly luciferase (FFL) activities were normalized by Renilla (RL) activities.

### Bioinformatics

Public tumor microarray databases were obtained from Gene Expression Omnibus (accession numbers GSE45436, GSE55092, GSE36915, GSE96851, GSE38941) and R software (https://bioconductor.org/biocLite.R) to analyze the expression of CENPM and miR-1270. The mRNA RNA-seq data and clinical data related to CENPM of HCC as well as Kaplan-Meier plots presenting overall survival and disease free survival of HCC patients were downloaded from The Cancer Genome Atlas (TCGA) liver cancer dataset (LIHC) and GEPIA (http://gepia.cancer-pku.cn). Single-gene gene set enrichment analysis (GSEA) analysis was performed to find the significant pathways between low expression and high expression group of CENPM. TargetSan (http://www.targetscan.org/), miRDB (http://mirdb.org/miRDB/), miRNA.org. (http://www.microrna.org/microrna/home.do/) were used for identifying miRNAs involved in the post-transcriptional regulation of CENPM.

### Statistical analysis

All data in this study were showed as the means ± standard deviation (MEAN ± SD). The significance of the changes among tumor and normal group was judged by Student’s t test with GraphPad Prism 6 software (La Jolla, USA) and the value of *P* < 0.05 was considered significant. Kaplan-Meier method and Cox’s proportional hazards regression model were used to calculate overall survivals, and the differences were analyzed by a log-rank test.

## Results

### The expression of CENPM in different gene expression omnibus (GEO) and TCGA datasets

**Fig. 1:**
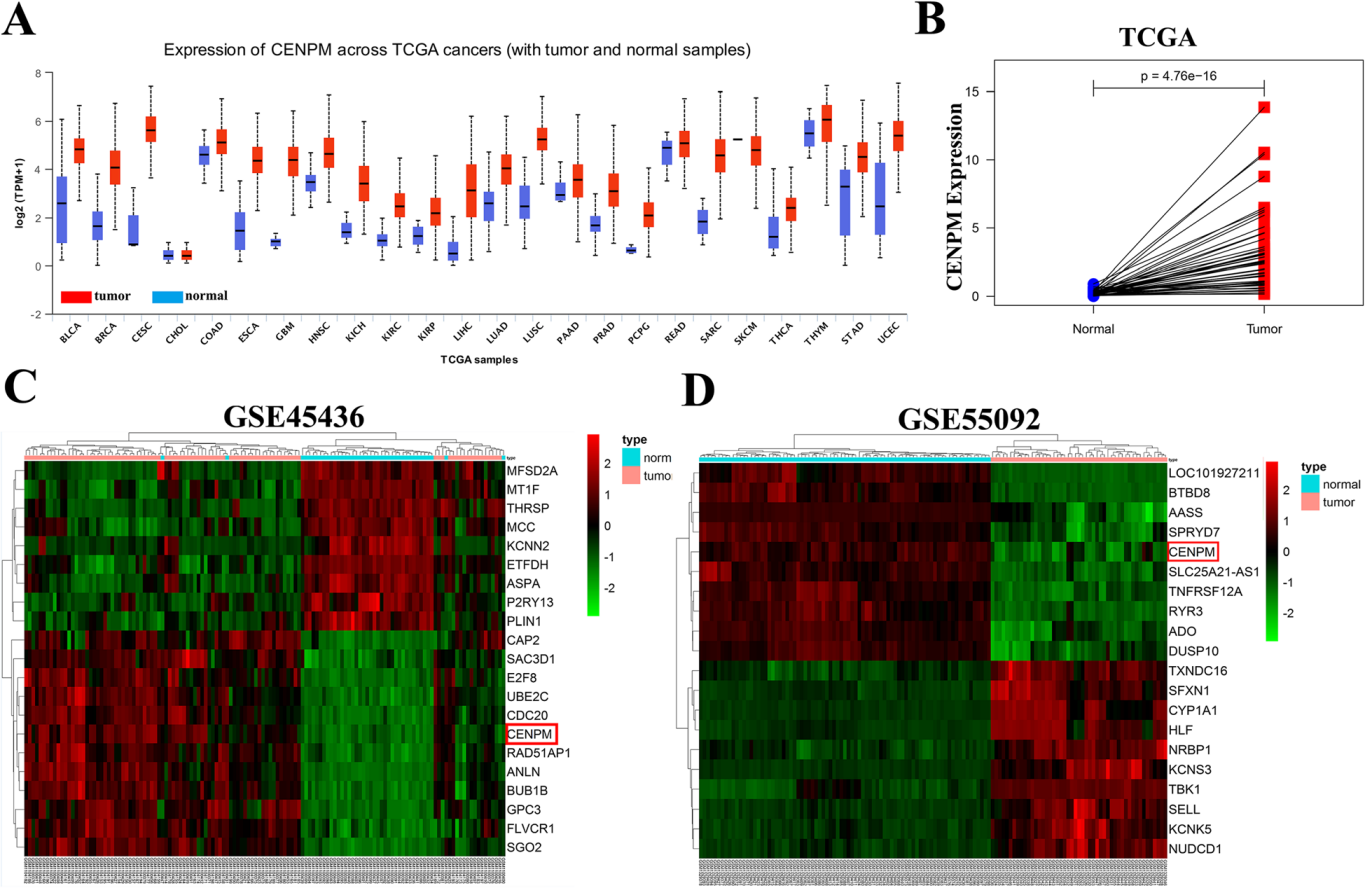
The expression of CENPM in different GEO and TCGA datasets. **a** The mRNA level of CENPM in 24 kinds of tumor types from TCGA. **b** CENPM expression in 50 paired HCC group downloaded from TCGA RNA-seq datasets. **c, d** Heatmaps of top 20 differential expressed mRNAs identified from GSE45436 and GSE55092

We first checked the mRNA level of CENPM in 24 kinds of tumor types from TCGA to figure out the CENPM whether was a oncogene or a tumor suppressor, and found that the expression of CENPM was almost higher in tumor group than corresponding normal group (Fig. 1a). Besides, CENPM was significantly over-expressed in 50 paired HCC group downloaded from TCGA whole transcriptome sequencing (RNA-seq) dataset (Fig. 1b). And heatmaps from GSE45436 and GSE55092 confirmed CENPM up-regulation in HCC group (Fig. 1c-d).

### CENPM was remarkly over-expressed in HCC and associated with poor prognosis

**Fig. 2:**
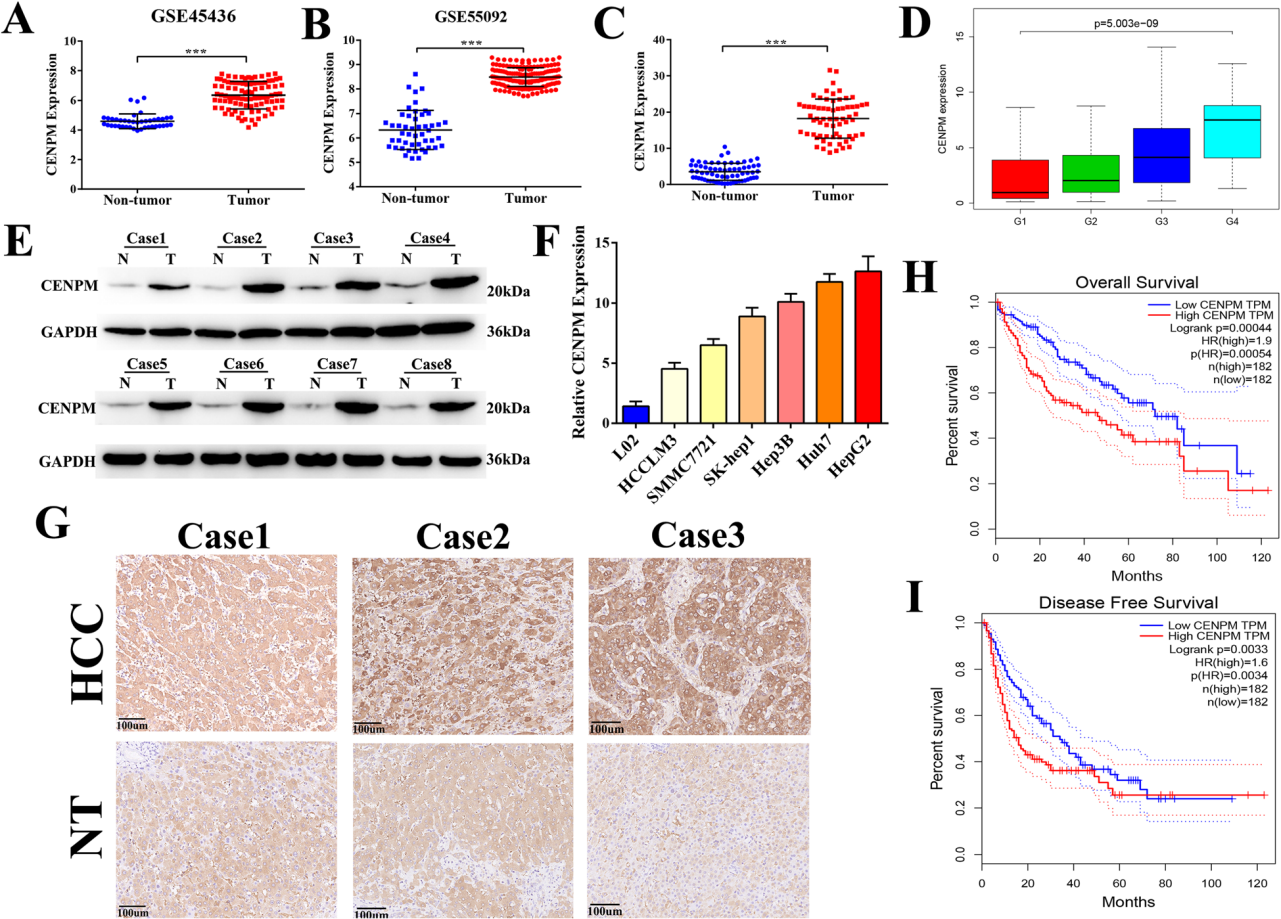
CENPM was overexpressed in HCC and significantly associated with poor prognosis. **a**, **b** CENPM was upregulated in tumor compared with normal liver tissues in HCC samples from GSE45436 and GSE55092. **c** Expression levels of CENPM in HCC tissues were higher than corresponding non-tumor tissues via qRT-PCR of 68 pairs of HCC samples. **d** Differential expression of CENPM in tumor grades. **e** CENPM protein level in 8 pairs of human clinical HCC tumor samples and adajacent non-tumor specimens. **f** CENPM relative expression in HCC cell lines (SMMC7721, Hep3B, HepG2, HCCLM3, Huh7 and SK-Hep1) compared with the immortalized normal human hepatic cell (L02)**. g** Typical images of immunohistochemistry (IHC) in 18 pairs of HCC tissues showing the protein expression of CENPM in HCC and adjacent nontumor tissues. **h, i** Kaplan-Meier analysis of overall survival and disease free survival in 364 HCC patients from the TCGA dataset. ***P < 0.001,T-Tumor; NT-Nontumor

As mentioned previously, the expression of CENPM from GEO database was significantly up-regulated in tumor group compared with normal group (Fig. 2a-b). Then 68 paired HCC samples and matching adjacent non-tumor tissues obtained from Zhongnan Hospital of Wuhan University were used for examining the mRNA level of CENPM via qRT-PCR, and the results were consistent with GEO databases and TCGA data analysis (Fig. 2c), and also confirmed by western blotting (Fig. 2e). Besides, the expression of CENPM was closely related to tumor grades of HCC (Fig. 2d). Moreover, the high protein level of CENPM in 18 pairs of HCC specimens was further verified by IHC (Fig. 2g). As shown in the Fig. 2f, the CENPM expression was higher in hepatoma cells than in the immortalized liver cell line L02 (Fig. 2f). Additionally, Kaplan–Meier and Cox’s proportional hazards regression model survival analysis revealed that patients with high expression levels of CENPM had shorter overall survival(Fig. 2h-i, Table 1).

**Table 1:** Univariate and multivariate analyses of clinicopathological characteristics, and CENPM with overall survival in TCGA LIHC cohort

	Univariate analysis		Multivariate analysis	
Characteristics	HR (95% CI)	*p*-value	HR (95% CI)	*p*-value
Gender				
(male vs female)	0.862 (0.595–1.249)	0.434		
Age, years				
(≥median vs. < median)	1.012 (0.998–1.027)	0.102		
Risk factors				
(alcohol consumption vs non-alcohol consumption)	1.114 (0.828–1.499)	0.476		
Histologic grade				
(G1 + G2 vs G3 + G4)	1.215 (0.879–1.414)	0.0389*		
Clinical stage				
(stage I + II vs stage III + IV)	1.381 (1.149–1.660)	0.000*		
Pathologic T stage (present vs. absent)	1.673 (1.393–2.010)	0.000*	1.645 (1.192–2.269)	0.002*
Pathologic N stage (present vs. absent)	0.140 (0.045–0.442)	0.007*	0.169 (0.038–0.740)	0.018*
Pathologic M stage (present vs. absent)	0.089 (0.0221–0.362)	0.000*	0.335 (0.10–1.126)	0.070*
CENPM				
(≥median vs. < median)	1.043 (1.014–1.073)	0.003*	1.025 (0.799–1.057)	0.032*

*HR*, Hazard ratio, *CI*, Confidence interval. Bold italics indicate statistically significant values (*p < 0.05)

Above all, we considered CENPM was over-expressed in HCC and associated with poor survival and disease progression.

### CENPM knock-down inhibited hepotoma cell proliferation, migration as well as invasion

**Fig. 3:**
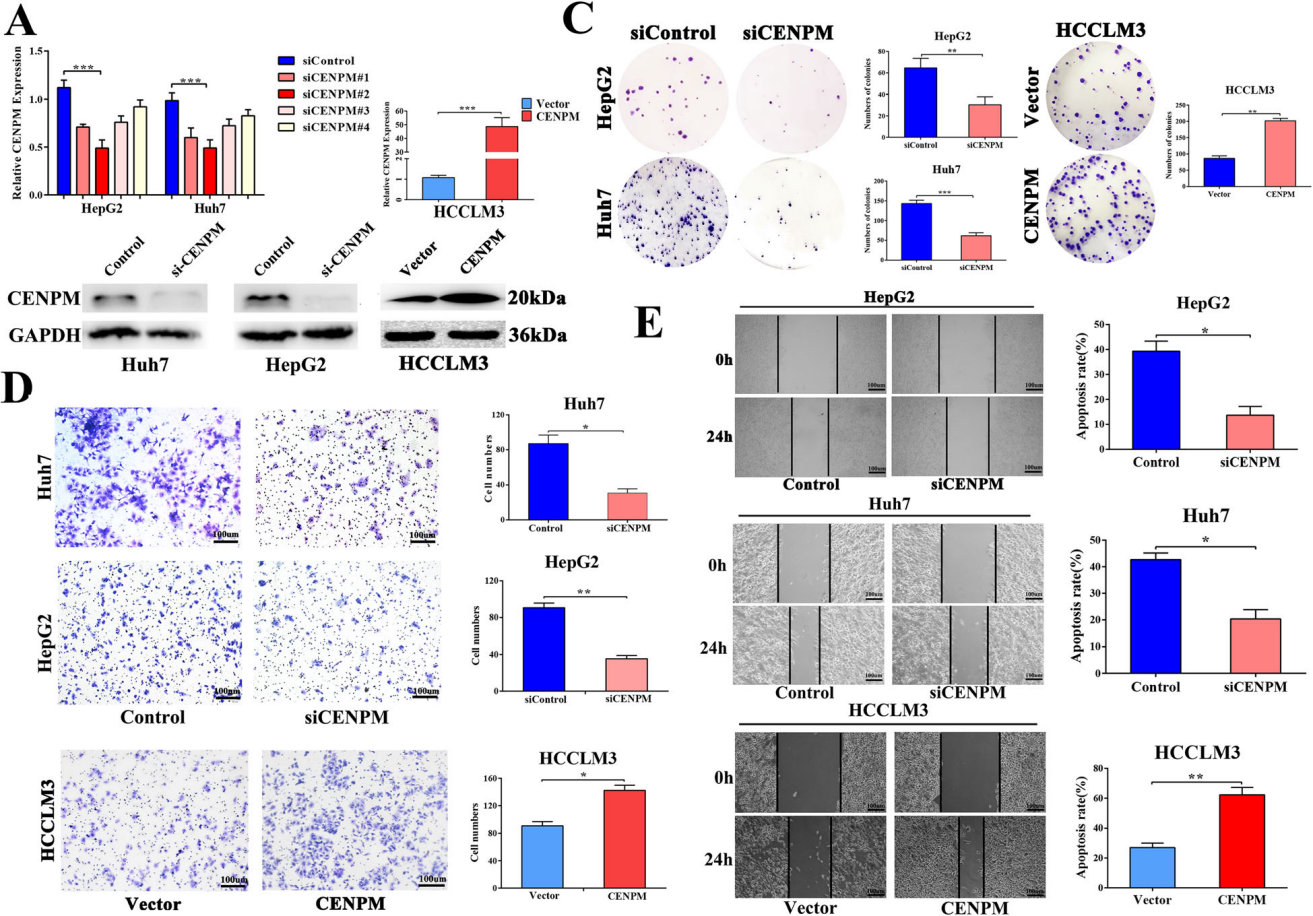
Knock-down CENPM inhibited HCC proliferation, cell invasion and cell migration in vitro. **a**, **b** CENPM knockdown efficiency and overexpression efficiency were confirmed by qPCR and western blotting in HepG2, Huh7 and HCCLM3 cell lines. **c** Colony formation assay showed that CENPM suppressed or promoted cell proliferation both in CENPM-knockdown HepG2 and Huh7 cell lines or CENPM overexpressing HCCLM3 cell line. **d, e** Downregulation of CENPM in HepG2 and Huh7 cell lines inhibited cell invasion and migration and CENPM overexpression in HCCLM3 reversed these results**.** *P < 0.05, **P < 0.01, ***P < 0.001

To explore the potential biological function of CENPM, we selected Huh7 and HepG2 cells as knockdown models of CENPM and HCCLM3 as an over-expression model according to the CENPM expression patterns (Fig. 2f). Four siRNAs (Designated #1-#4) and plasmids were chosen to transfect into the cells and the knockdown as well as overexpression effectiveness were evaluated by qRT-PCR(Fig. 3a) and western blotting (Fig. 3b). Then cell proliferative capacity was examined in Huh7 and HepG2 cells by colonies formation assay, and the results indicated that downregulation of CENPM significantly declined colony-forming efficiency (Fig. 3c, Additional file 2: Figure S1A), subsequent transwell assay and wound healing assay proved that down-regulated CENPM could suppress the migratory and invasive capability in Huh7 and HepG2 cells (Fig. 3d-e), we also confirmed above results in HCCLM3 cells transfected with CENPM plasmids. Moreover, we examined EMT-related markers by western blotting (Additional file 2: Figure S1B), and we found knockdown CENPM could increase expression of epithelial marker (E-cadherin), downregulate mesenchymal markers (N-cadherin, vimentin and fibronectin), suggesting that CENPM may be involved in promoting metastasis of HCC via EMT.

### CENPM low expression arrested cell cycle and promoted apoptosis of hepatoma cells

**Fig. 4:**
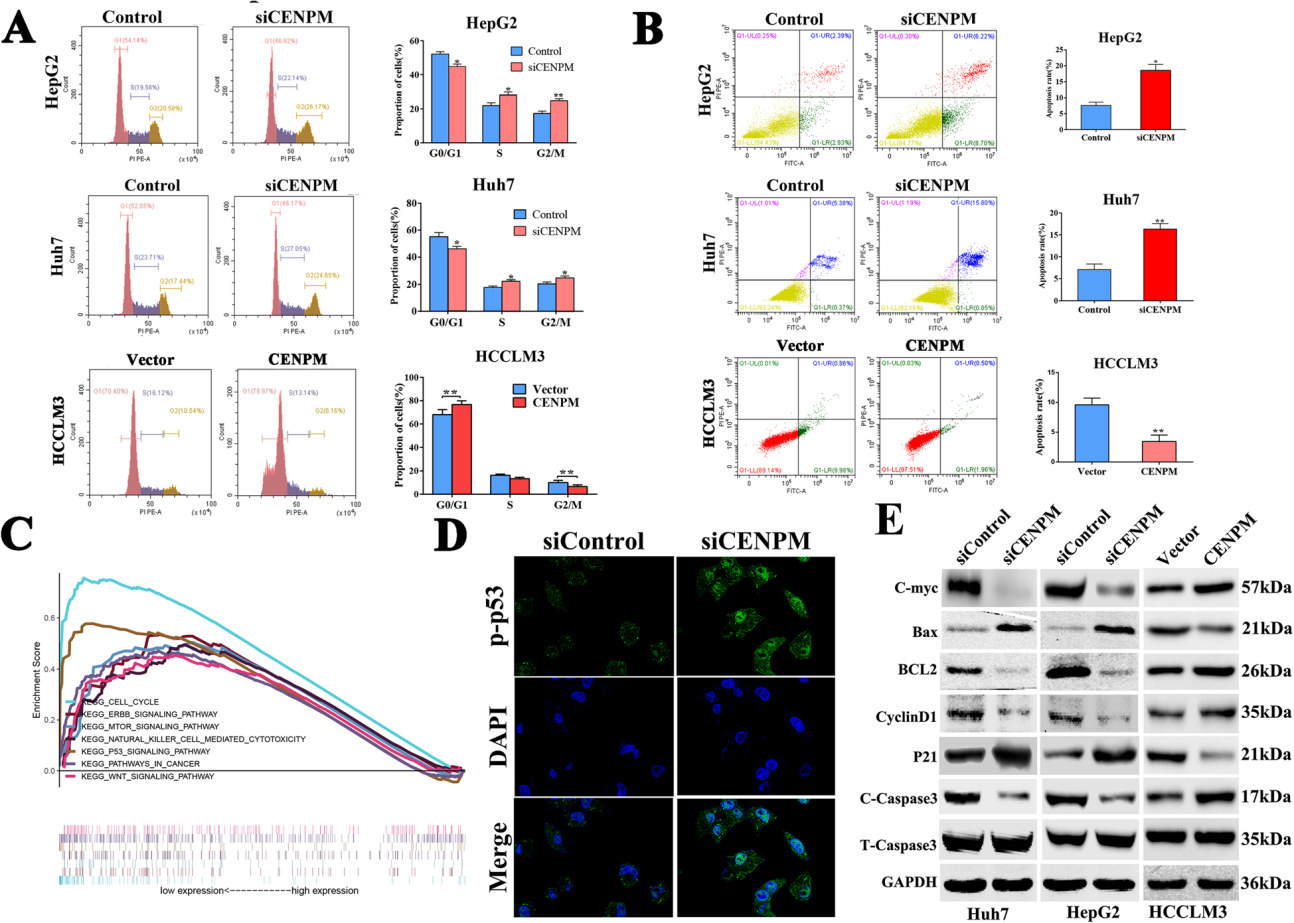
Depletion of CENPM arrested cell cycle and induced cell apoptosis. **a** FACS analysis showing significant increased or decreased in the proportion of cells in G2/M phase, respectively, when CENPM was knockdown in Huh7 and HepG2 cell or overexpressed in HCCLM3 cells. **b** Silencing CENPM promoted cell apoptosis in Huh7 and HepG2 cell lines or inhibited cell apoptosis in HCCLM3 cells. **c** Representative signal pathways of CENPM single-gene GSEA analysis. **d** Immunofluorescence images showed that CENPM downregulation promoted expression level of nuclear phosphorylated P53. **e** Western blotting analysis of C-myc, Bcl2, CyclinD1, Bax, P21, cleaved-caspase3 in CENPM-depleted Huh7 and HepG2 cell lines or CENPM-overexpressed HCCLM3 cells. *P < 0.05, **P < 0.01

Previous researches had proved that CENPM involved in regulating cell cycle [21, 22]. And our study confirmed low-expression of CENPM increased the proportion of cells in G2/M phase, however,decreased G0/G1 accordingly following CENPM knockdown in both Huh7 and HepG2 cell lines (Fig. 4a), besides, we also detected that silencing CENPM in cell lines mentioned above could induce more apoptotic cells (Fig. 4b), while the opposite results were attained when CENPM was overexpressed. Subsequent single-gene GSEA analysis confirmed low expression of CENPM was linked to the P53 signal pathway, cell cycle signal pathway and so on (Fig. 4c). As shown in Fig. 4d, the immunofluorescence suggested that expression of phosphorylated P53 located in the cell nucleus increased in CENPM knock-down group. And western blotting (Fig. 4e) also confirmed that CENPM was tightly associated with P53-mediated cell cycle and apoptosis. Silencing CENPM reduced the expression of C-myc, Bcl2, CyclinD1, while increased protein level of Bax, P21, C-caspase3, however, overexpressing CENPM in HCCLM3 cells reversed above results.

### Depletion of CENPM inhibited cell proliferation and cell apoptosis in vivo

**Fig. 5:**
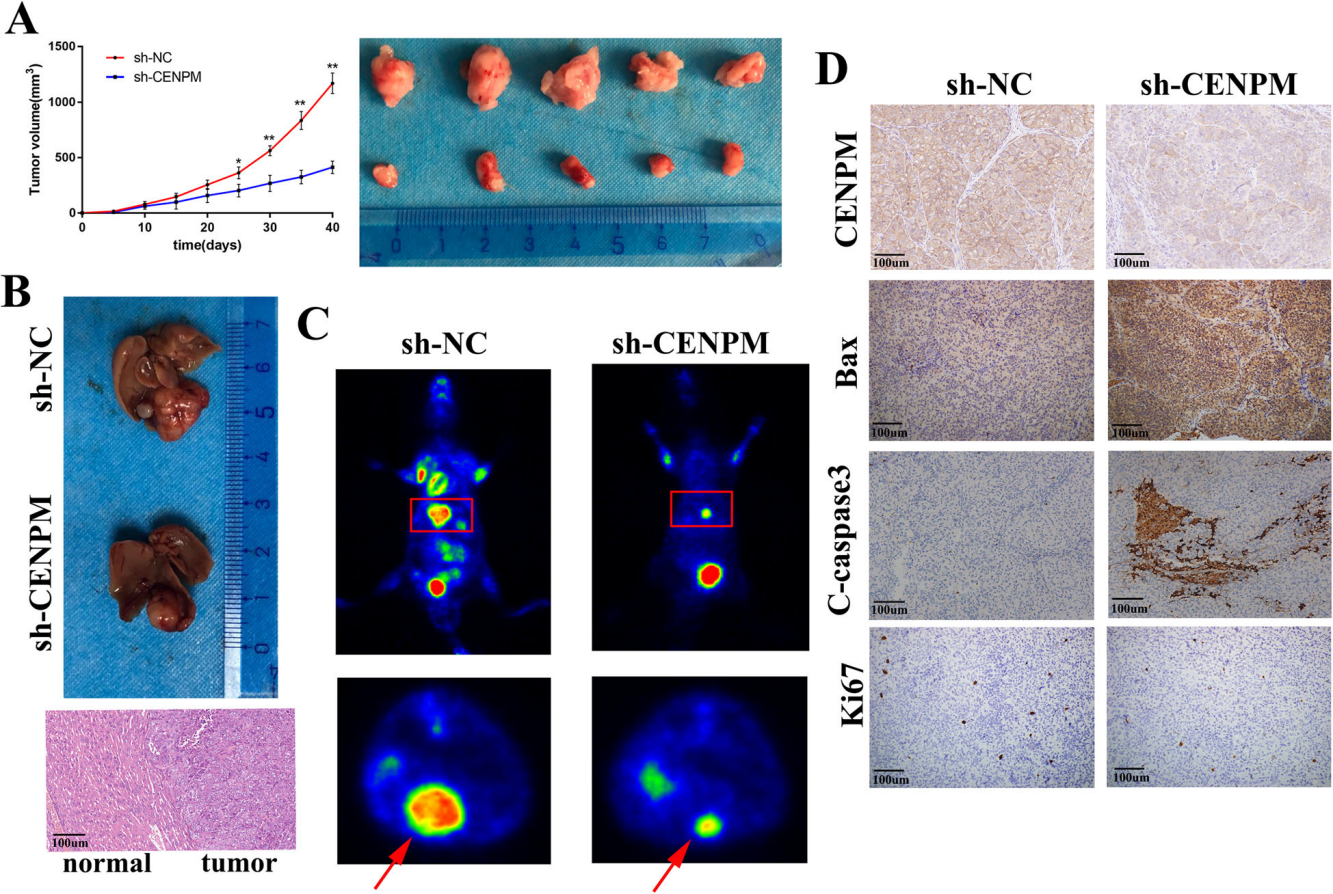
The carcinogenic effect of CENPM in vivo. **a** Representative images of tumor volume and tumors removed from nude mice implanted with shRNA-CENPM transfected HCCLM3 cells (*n* = 5) or control cells (*n* = 5). **b, c** Characteristic images of an orthotopic transplantation tumor model of HCC in nude mice,HE staining and PET/CT. **d** Xenografts were fixed, embedded in paraffin and stained with CENPM, Bax, cleaved caspase 3, Ki67 antibodies for analysis of tumor growth and apoptosis. **P < 0.01

To gain a deeper acquaintance of carcinogenic function about CENPM, we first conducted subcutaneous xenograft experiments in nude mice, HCCLM3 cells with shRNA-CENPM (sh-CENPM) and shRNA-NC (sh-NC) were injected into the armpit of male nude mice, the CENPM silencing efficiency in HCCLM3 cells (Additional file 3: Figure S2A) was confirmed to be significant. And it turned out that stable knockdown of CENPM significantly repressed tumor growth (Fig. 5a) and markedly decreased positivity for CENPM and Ki67, however, increased the expression of Bax and C-caspase3 (Fig. 5d). Then we further established an orthotopic transplantation tumor model of HCC in nude mice to confirm oncogenic role of CENPM, and after 6 weeks, PET/CT operated by Union Hospital PET Center of Tongji Medical College (Wuhan, China) was used for the measurement of liver lesions. According to PET/CT images results, the nude mice bearing sh-CENPM can block liver tumor growth (Fig. 5c). The liver dissection results in mice were accordance with PET/CT results, and H&E staining further confirmed liver tumor lesions in mice (Fig. 5b).

### HBx contributed to the upregulation of CENPM by repressing miR1270 in HCC cells

**Fig. 6:**
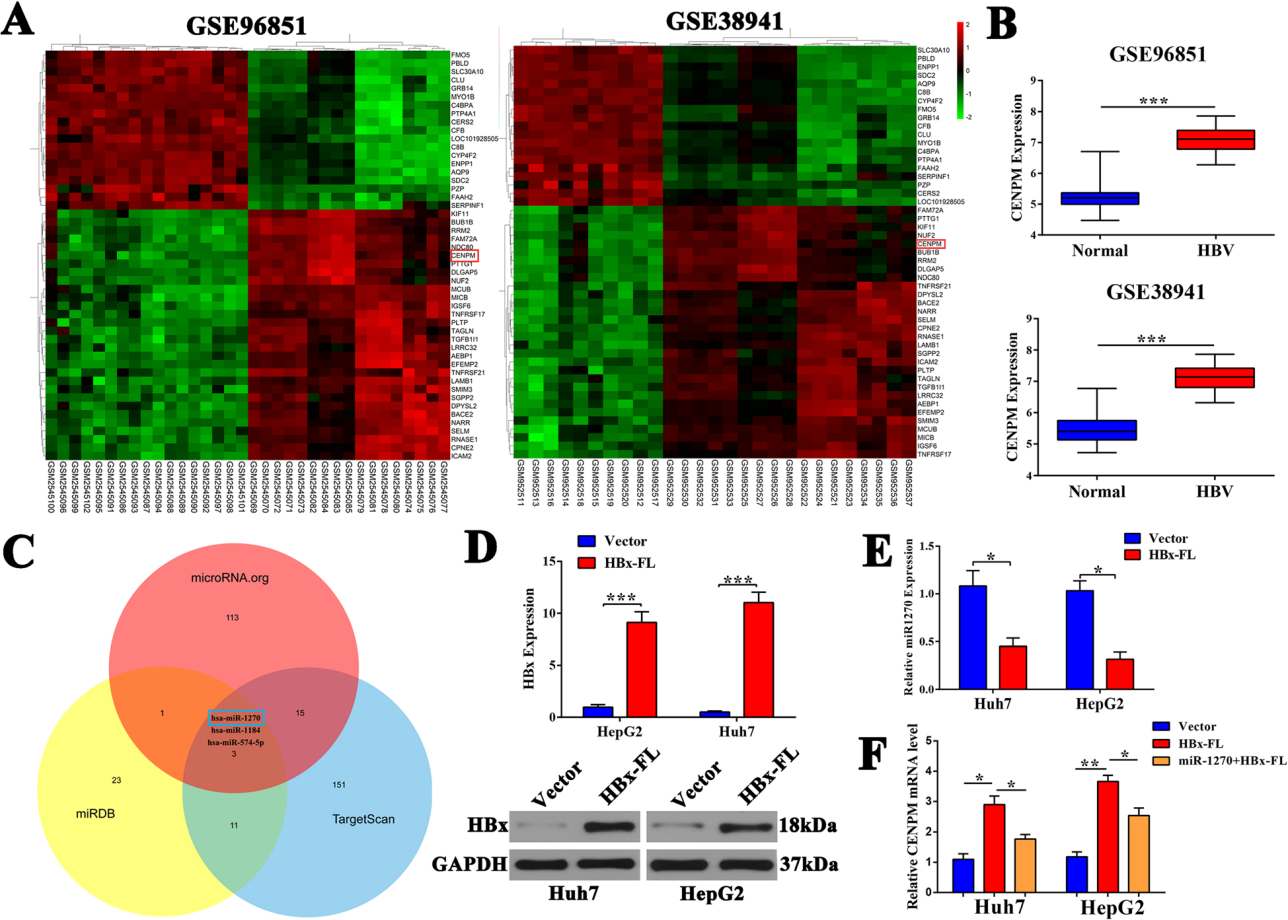
HBx upregulated CENPM by targeting miR1270. **a** The heatmaps of partial differential expressed genes in GSE96851 and GSE38941. **b** CENPM was up-regulated in HBV-related liver tissues compared with normal tissues in GSE96851 and GSE38941. **c** miR-1270 was recognized as a post-transcriptional regulator of CENPM by microRNA target prediction programs. **d** Western blotting showed HBx stably expressed in Huh7 and HepG2 cell lines**. e** miR-1270 was downregulated by RT-qPCR in HBx stably expressing Huh7 and HepG2 cell lines. **f** Transfection of HBx resulted in upregulation of CENPM through miR-1270, as determined by PCR. *P < 0.05, **P < 0.01

Chronic hepatitis B virus (HBV) infection is a leading cause of liver cancer [7]. We speculated whether CENPM expression was modulated by HBV. So we downloaded the HBV-related GEO datasets. The heatmaps and histograms of GSE96891 and GSE38941 confirmed CENPM was overexpressed in HBV-related liver tissues compared with normal tissues (Fig. 6a-b). However, the relationship between HBV/HBx and abnormal CENPM expression in HCC hadn’t been fully established. In addition, abundant literature pointed out that microRNAs were involved in posttranscriptional regulation of mRNAs [23, 24], so we used microRNA.org, miRDB, TargetScan to predict potential microRNA binding sites in the 3’UTR of CENPM. As shown in Fig. 6c, miR-1270 was identified. Recent research reported hepatitis B virus X protein up-regulated Alpha-fetoprotein to promote HCC by targeting miR-1236 and miR-1270 [25], so we first established HBx stably expressing Huh7 and HepG2 cell lines (Fig. 6d), and high HBx overexpression efficiency in Huh7 and HepG2 cells were demonstrated in Additional file 3: Figure S2B-C. Then subsequent RT-qPCR confirmed that HBx transfection could lead to apparent decrement of miRNA 1270 (Fig. 6e), and HBx overexpression can partially offset the down-regulation of miRNA to CENPM (Fig. 6f).

### Over-expression of miR-1270 in HCC leaded to CENPM down regulation

**Fig. 7:**
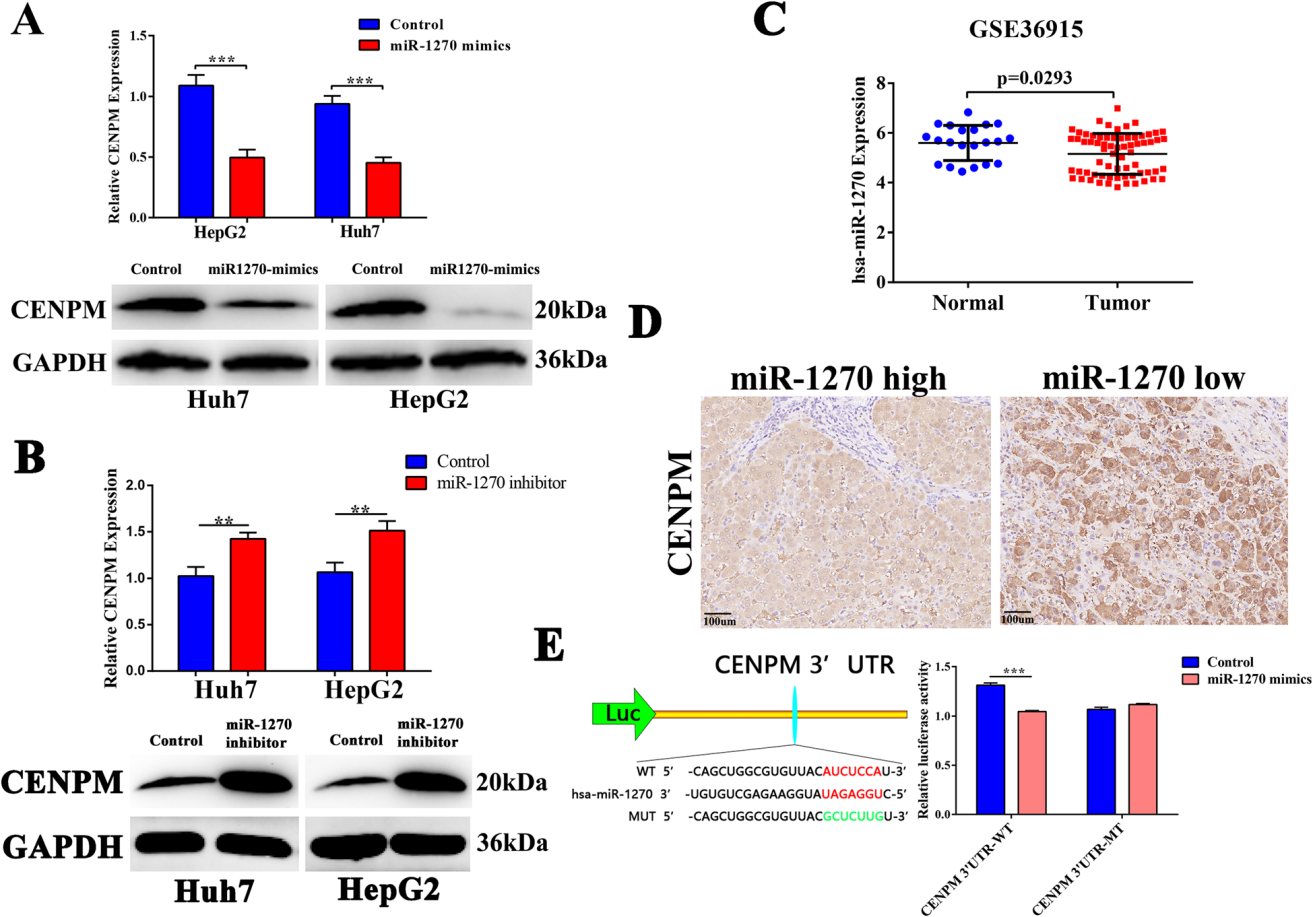
miR-1270 negatively regulated CENPM expression in human HCC. **a** Overexpression of miR-1270 declined CENPM mRNA and protein expression level in Huh7 and HepG2 cells. **b** CENPM mRNA and protein level was increased after inhibition of miR-1270. **c** miR-1270 was downregulated in HCC in GSE36915. **d** IHC of CENPM in HCC samples showed overexpression of miR-1270 reduced CENPM protein levels. **e** Luciferase reporter assay demonstrated that upregulation of miR-1270 decreased luciferase activity of wild-type CENPM 3′UTR but not that of the mutant. *P < 0.05, **P < 0.01, ***P < 0.001

RTq-PCR and western blotting (Fig. 7a-b) indicated that transfection of miR-1270 mimics or inhibitors in Huh7 and HepG2 cells could inhibit or increase the mRNA and protein expression of CENPM, respectively. And RNA-seq data from GSE36915 demonstrated that CENPM was down-regulated in HCC group (Fig. 7c). IHC also verified that miR-1270 was down regulated in CENPM high-expressed specimens (Fig. 7d). To further make sure the binding sites between miRNA1270 and CENPM, luciferase reporter vectors containing the wild type(WT) and mutant (MUT) miR-1270 binding sequences of the CENPM were constructed. The luciferase reporter assay demonstrated that upregulation of miR-1270 dramatically weakened the luciferase activity of WT luciferase vector, while no significant change in the MUT luciferase vector (Fig. 7e). Taken together, these results proved that miR-1270 was a negative regulator and involved in post-transcriptional regulation of CENPM.

## Discussion

HCC is an aggressive cancer resulting in major morbidity and mortality in China [26], and current therapies including surgical resection [27], liver transplantation [28], radiofrequency ablation (RFA) [29], transcatheter arterial chemoembolization (TACE) are effective but of limited use [30], the median survival of HCC patients is only 6 months [31], therefore, it is very urgent to figure out the regulatory mechanisms of signal pathways involved in HCC as well as to find out a novel significative biomarker of HCC.

In this study, we first put forward that CENPM was up-regulated in HCC samples, which was correlative with a poor prognosis of HCC, besides, bioinformatics prediction demonstrated low expression of CENPM was related to P53 signal pathway and cell cycle pathway, subsequent experimental findings confirmed that.

CENPM (centromere protein M), is an original gene that was firstly recognized for its over expression in b-catenin transformed mouse mammary tissue [12, 15], which is a component of the CENPA-NAC (nucleosome-associated) complex, and this complex plays a crucial role in assembly of kinetochores proteins, mitotic progression and chromosome segregation during cell division [14]. And it is well known that the mechanism of chemotherapeutic drugs mainly involves direct or indirect destruction of DNA [32], acting on tubulin [33], an inhibitor of protein synthesis [34]. CENPM may act as a potential target of chemotherapeutics whose main role is preventing tubulin formation or promoting decomposition to inhibit cell mitosis, thereby enhancing the chemotherapeutic sensitivity of cancers. In addition, totally discovering the mechanism of chromosomal instability may provide new therapeutic targets for most solid tumors. And genes that potentially influence chromosome segregation are the most hopeful candidates. If CENPM overexpression is the mainspring of tumor progression, inhibition of its expression level in cancer cells might stop tumor proliferation. It had reported that micro-injection of antibodies to CENPA arrested HeLa cells in interphase [17]. Therefore, it will be promising to design an anticancer drug targeting the specific chemical structure of CENPM. Moreover, CENPM can encode a human minor histocompatibility antigen expressed by tumor cells [35, 36], it had reported that CENPM had an association with tumor progression [22, 37]. Other components of CENPA-NAC, including CENPA, CENPF, and CENPH, were all described to be correlated with tumors, for example, overexpression and mistargeting of CENP-A could contribute to human primary colorectal cancer [17], CENPF had been linked to a poor prognosis in breast cancer, colorectal gastrointestinal stromal tumors and nasopharyngeal carcinoma patients [19]. Low-expression of CENPM was related to a better overall survival rate in bladder cancer [38]. Nowadays, although CENPM has been extensively documented, how CENPM participates in oncogenesis of HCC is still in its initial stages.

In our research, we showed CENPM was over-expressed in HCC specimens compared to the corresponding para-carcinoma tissues, which was in accordance with bioinformatics, and CENPM was also discovered to be up-regulated in six HCC cell lines. Subsequent Kaplan-Meier and Cox’s proportional hazards regression model analysis obtained from TCGA-LIHC further proved that CENPM was tightly linked to poor overall survival in HCC patients. Then we explored the mechanisms of CENPM-mediated regulations on hepatoma cells and detected that depletion of CENPM dramatically suppressed cellular proliferation, cell invasion and cell migratory capacity. Additionally, we established xenograft model and orthotopic HCC mouse model to further verify that CENPM promoted tumor growth. Previous study reported CENPM might participate in the cell cycle [39, 40], and to better clarify the biological functions of CENPM, single-gene GSEA was conduct and Kyoto Encyclopedia of Genes and Genomes (KEGG) enrichment analysis indicated low expression of CENPM was indeed enriched in P53 signaling pathway, cell cycle and so on. P53 was regarded as a transcription factor that promoted and blocked target genes participated in cell cycle, apoptosis, programmed necrosis, metabolism, stem cell homeostasis, angiogenesis, senescence and immune regulation [41, 42]. Our flow cytometry analysis and IF of p-P53 also confirmed the GSEA predictions, and protein levels of P53 related markers were also changed owing to the CENPM knockdown.

Accumulated evidence demonstrated abnormal expression of microRNAs were deeply associated with post-transcriptional regulations of target genes [21, 43, 44]. It had described high expression of miR-195-5p can down regulate human yep-associated protein (YAP) mRNA, so as to inhibit tumor development in cancers [45]. In our study, we found that miR-1270 was a negative regulator of CENPM. The miR-1270 specifically targeted the binding sites at the 3′-UTR of the CENPM mRNA and possibly contributed to low expression of CENPM. A dual luciferase reporter assay also supported this conclusion. Additionally, we further revealed that HBx can lead to a significant increment of CENPM expression by targeting miR-1270.

## Conclusions

We first discovered CENPM was oncogene in HCC, the high expression of which was correlative with a poor prognosis. We found CENPM promoted cell proliferation, cell invasion and migration. Besides, CENPM can promote cell cycle progress and inhibit apoptosis by taking part in the regulation of P53 signal pathway. Furthermore, miR-1270 served as a suppressor was regarded to be involved in post-transcriptional regulation of CENPM, and HBx can inhibit miR-1270 expression to upregulate CENPM to promote HCC. Taken together, our discoveries further evidenced that CENPM can be as a prognostic biomarker and a new therapeutic target in HCC.

## Supplementary information

**Additional file 1: Tables S1-S2. (Table S1)** Primer sequences and target sequences. **(Table S2)** Antibodies used in this study.

**Additional file 2: Figure S1. (Figure S1A)** Colony formation assay in CENPM-knockdown HepG2 and Hu7 cells and CENPM overexpressed HCCLM3 cell lines. **(Figure S1B)** Western blotting assay examined expression levels of EMT markers (E-cadherin, N-cadherin, vimentin, and fibronectin) in HepG2, Huh7 and HCCLM3 cells after depletion or overexpression of CENPM.

**Additional file 3: Figure S2. (Figure S2A)** Representative image of transfected HCCLM3 cells were captured with a fluorescence microscope, and CENPM silencing efficiency in HCCLM3 cells. **(Figure S2B)** Representative images of HBx stably expressing Huh7 and HepG2 cell lines.

## Data Availability

The authors declare that all data and materials are available on request.

## Abbreviations

3′UTR: 3′ untranslated region
AFP: Alpha-fetoprotein
CENPM: Centromere protein M
GEO: Gene Expression Omnibus
GSEA: Gene set enrichment analysis
HBV: Hepatitis B virus
HBx: Hepatitis B Virus X Protein
HBx-FL: Lentivirus carrying full length HBx
HCC: Hepatocellular carcinoma
HE: Hematoxylin–eosin
IF: Immunofluorescence
IHC: Immunohistochemistry
LIHC: Liver Hepatocellular Carcinoma
PANE1: Proliferation associated nuclear element 1
PVDF: Polyvinylidene difluoride
RFA: Radiofrequency ablation
RT-qPCR: Real-time quantitative polymerase chain reaction
SDS-PAGE: Sodium dodecyl sulfate–polyacrylamide
shRNA-CENPM: Small hairpin RNA expression vector targeting human CENPM gene
shRNA-NC: Scrambled shRNA
siRNA: small interfering RNAs
TACE: Transcatheter arterial chemoembolization
TCGA: The Cancer Genome Atlas
