# Supplementary material for: Upregulation of CENPM promotes hepatocarcinogenesis through mutiple mechanisms
Source: J Exp Clin Cancer Res. 2019 Nov 8;38:458. doi: 10.1186/s13046-019-1444-0 (PMC6839178; doi:10.1186/s13046-019-1444-0)
Supplement: Supplementary file 1 — Additional file 1: Tables S1-S2. (Table S1) Primer sequences and target sequences. (Table S2) Antibodies used in this study. [file 13046_2019_1444_MOESM1_ESM.docx]

**Supplementary Tables**

**Supplementary Table 1. Primer sequences and target sequences**

| **Name** | **Sequence or target sequence** |
| --- | --- |
| CENPM-F | 5′-CCAGAACACAGAGGAGTC-3′ |
| CENPM-R | 5′-CAGGTCACAGTAGAGCAG-3′ |
| GAPDH-F | 5′-GTCTCCTCTGACTTCAACAGCG-3′ |
| GAPDH-R | 5′-ACCACCCTGTTGCTGTAGCCAA-3′ |
| miR-1270-F | 5'-CTGGAGATATGGAAGAGCT-3' |
| miR-1270-R | 5'- CAGTGCGTGTCGTGGAGT-3' |
| U6-F | 5'-CTCGCTTCGGCAGCACA-3' |
| U6-R | 5'-AACGCTTCACGAATTTGCGT-3' |
| siCENPM #1 | 5'- GAATTGACCTGATCGTGTT-3' |
| siCENPM #2 | 5'- CCTGATCGTGTTTGTGGTT-3' |
| siCENPM #3 | 5'- GATCGTGTTTGTGGTTAAT -3' |
| siCENPM #4 | 5'-TGGTTAATCTTCACAGCAA -3' |
| siControl | 5'-UUCUCCGAACGUGUCACGUTT-3' |
| ShRNA-CENPM | 5'-GATCGTGTTTGTGGTTAATCGAAATTAACCAC |
|  | AAACACGATCTTTTT-3' |
| MiR-1270 mimics | 5′-CUGGAGAUAUGGAAGAGCUGUGU-3′ |
|  | 5′-ACACAGCUCUUCCAUAUCUCCAG-3′ |
| Negative control | 5′-UUCUCCGAACGUGUCACGUTT-3′ |
| HBx-F | 5′-ACGUGACACGUUCGGAGAATT-3′  5′-TATGTCGACATGGCATCAATGCAGAAGCTGATC |
| HBx(full-length)-R | TCAGAGGAGGACCTGATGGCTGCTAGGCTGTGC-3′  5′-TATCCGCGGGGCAGAGGGGAAAAAGTTGTTG-3′ |
|  |  |

**Supplementary Table 2. Antibodies used in this study**

| **Antibody** | **WB** | **IHC** | **IF** | **Specificity** | **Company** |
| --- | --- | --- | --- | --- | --- |
| CENPM(abs132709) | 1:1000 | 1:100 |  | Rabbit polyclonal | Absin |
| C-myc(ab39688) | 1:1000 |  |  | Rabbit monoclonal | Abcam |
| Bax(ab53154) | 1:1000 | 1:100 |  | Rabbit monoclonal | Abcam |
| Bcl2(ab59348) | 1:1000 |  |  | Rabbit monoclonal | Abcam |
| CyclinD1(#2978) | 1:1000 |  |  | Rabbit monoclonal | Cell Signaling Technology |
| P21(ab109520) | 1:2000 |  |  | Rabbit monoclonal | Abcam |
| Caspase3(32351) | 1:5000 |  |  | Rabbit monoclonal | Abcam |
| C-caspase3(ab2302) | 1:1000 | 1:50 |  | Rabbit monoclonal | Abcam |
| Phospho-p53(Ser15)(#9286) |  |  | 1:400 | Mouse monoclonal | Cell Signaling Technology |
| GAPDH(#10494-1-AP) | 1:5000 |  |  | Rabbit Polyclonal | Proteintech |
| Ki67( ab92742) |  | 1:200 |  | Rabbit monoclonal | Abcam |
