# Supplementary figures and images for: Upregulation of CENPM promotes hepatocarcinogenesis through mutiple mechanisms
Source: J Exp Clin Cancer Res. 2019 Nov 8;38:458. doi: 10.1186/s13046-019-1444-0 (PMC6839178; doi:10.1186/s13046-019-1444-0)

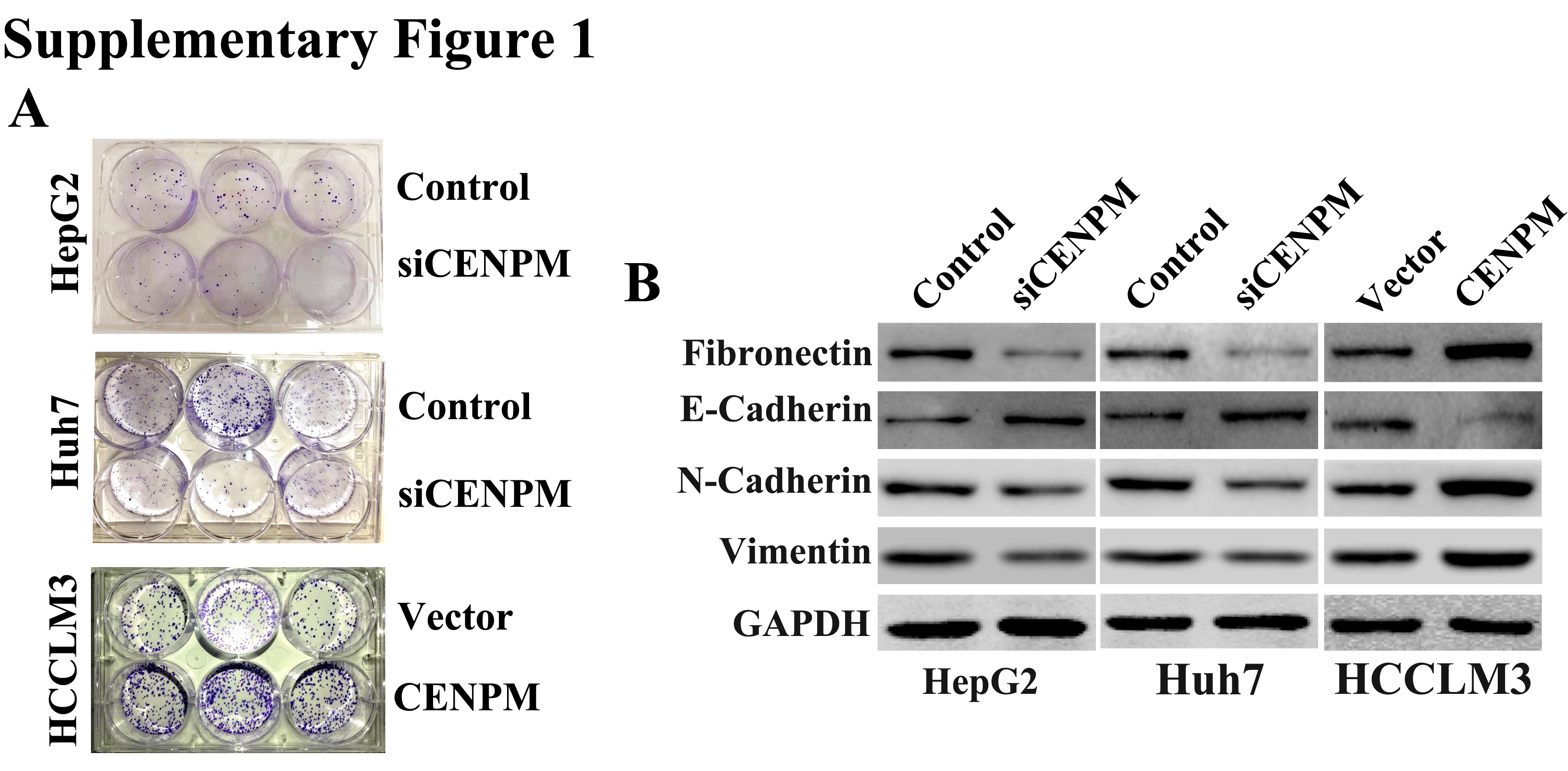

Supplement: Supplementary file 2 — Additional file 2: Figure S1. (Figure S1A) Colony formation assay in CENPM-knockdown HepG2 and Hu7 cells and CENPM overexpressed HCCLM3 cell lines. (Figure S1B) Western blotting assay examined expression levels of EMT markers (E-cadherin, N-cadherin, vimentin, and fibronectin) in HepG2, Huh7 and HCCLM3 cells after depletion or overexpression of CENPM. [file 13046_2019_1444_MOESM2_ESM.tif]

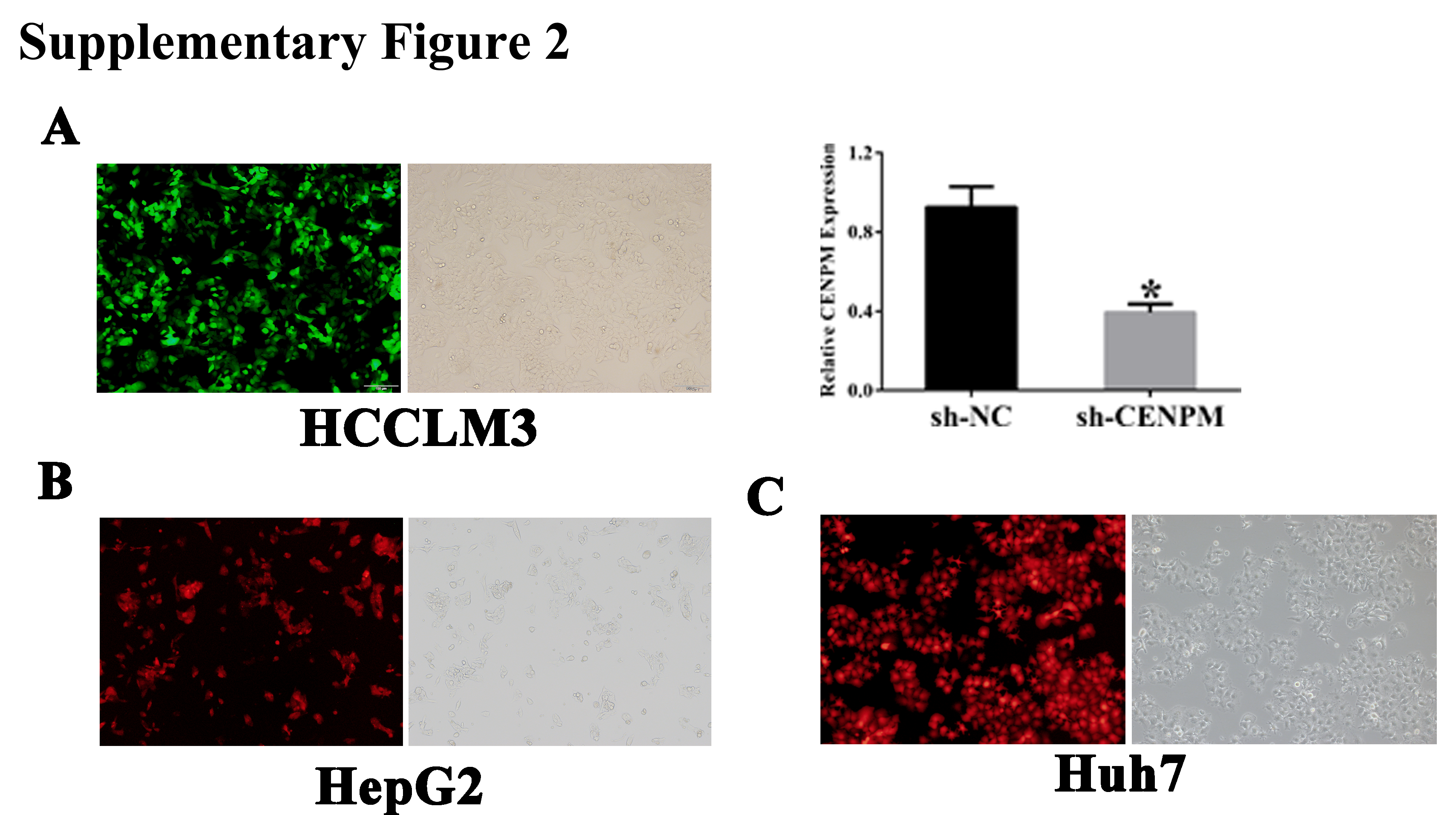

Supplement: Supplementary file 3 — Additional file 3: Figure S2. (Figure S2A) Representative image of transfected HCCLM3 cells were captured with a fluorescence microscope, and CENPM silencing efficiency in HCCLM3 cells. (Figure S2B) Representative images of HBx stably expressing Huh7 and HepG2 cell lines. [file 13046_2019_1444_MOESM3_ESM.tif]
